# The Medical Magazine

**Published:** 1892-09

**Authors:** 


*The Medical Magazine.* A Monthly Review of Medicine, Surgery and Allied Sciences. London: SOUTHWOOD, SMITH & CO. Vol. I., No. 1. July, 1892.

This is a specially welcome addition to our Exchange-list, inasmuch as it proposes to treat professional life and thought in a way barely touched by existing medical journals. It will offer no detail of clinical work, rightly believing that for this there are almost opportunities enough. But it will deal with medical questions on a broad and philosophic basis, and, if these are entrusted to writers of power and experience, its influence for good should be marked and far-reaching; for it is an undoubted fact that at present our profession has not attained in the public estimation the position which, as the custodian of the public health, it deserves.

Amongst the aims of such a publication as this should be the fostering of medical *belles-lettres*, for it is a sad reflection that in these days of rapid work and keen competition the medical man of high literary culture is fast disappearing, and it is rare to meet with medical communications, the style of which could be fairly described as attractive or even good. Doctors too often forget that "*omne tulit punctum qui miscuit utile dulci*," and so jeopardise the good effect of high-class scientific work.

With careful discrimination and experienced editorship there is a mission for this new magazine. We heartily wish it well, and strongly recommend our readers to get it and judge of it for themselves.

